# Correlation between circulating tumor cell levels and tumor response in patients with advanced hepatocellular carcinoma undergoing immunotherapy and molecular targeted therapy

**DOI:** 10.3389/fonc.2026.1917039

**Published:** 2026-09-29

**Authors:** Jiangmin Zhou, Huifang Xiong, Honghao Zhou, Wei Wang, Cheng Zhou

**Affiliations:** 1 Department of Hepatobiliary Surgery, Wuhan Hospital of Traditional Chinese and Western Medicine, Wuhan, China; 2 Department of Gastroenterology, People’s Hospital of Dongxihu District, Wuhan, China; 3 Department of Hepatic Surgery, Tongji Hospital, Tongji Medical College, Huazhong University of Science and Technology, Wuhan, China

**Keywords:** circulating tumor cells, hepatocellular carcinoma, immunotherapy, liquid biopsy, molecular targeted therapy, mRECIST, tumor treatment response

## Abstract

Circulating tumor cells (CTCs) are closely associated with the progression of hepatocellular carcinoma (HCC). However, the clinical value of CTC detection in patients receiving systemic therapy for advanced HCC remains to be fully elucidated. A total of 125 patients with advanced HCC who underwent systemic therapy were enrolled in this study. Five milliliters of peripheral blood were collected at five time points: before treatment initiation, and 1 month, 3 months, 6 months, and 1 year after treatment. CTCs were isolated and captured using the Isolation by Size of Epithelial Tumor Cells (ISET) method. Tumor response to systemic treatment was evaluated according to the modified Response Evaluation Criteria in Solid Tumors (mRECIST). We further analyzed the correlation between dynamic changes in peripheral blood CTC levels and tumor response to systemic therapy. The overall objective response rate (ORR) and disease control rate (DCR) were 39.2% (49/125) and 82.4% (103/125), respectively. Dynamic monitoring of CTCs revealed that CTC levels remained persistently high and gradually increased in patients with progressive disease (PD). In patients with complete response (CR) or partial response (PR), CTC levels decreased progressively during treatment and stayed at low levels thereafter. For patients with stable disease (SD), CTC counts began to decline 3 months after treatment but remained higher than those in patients with CR or PR. Baseline CTC levels were negatively correlated with treatment efficacy: patients with higher baseline CTC counts exhibited poorer tumor responses. Patients with baseline CTC counts < 15, CTC counts < 13 at 1 month post-treatment, and CTC counts < 10 at 3 months post-treatment were more likely to achieve an objective tumor response and obtained better therapeutic outcomes. Additionally, these patients had significantly longer progression-free survival (PFS) and overall survival (OS) compared with those who exceeded the above cut-off values. Peripheral blood CTC counts are correlated with tumor treatment response in patients with advanced HCC. Serial CTC detection serves as a valuable tool for evaluating the efficacy of systemic therapy in advanced HCC.

## Introduction

Hepatocellular carcinoma (HCC) is the predominant subtype of primary liver cancer, accounting for more than 90% of all liver cancer cases (1). Multiple risk factors contribute to HCC development, including chronic hepatitis B virus (HBV) and/or hepatitis C virus (HCV) infection, excessive alcohol consumption, non-alcoholic steatohepatitis (NASH), autoimmune liver diseases, drug-induced liver injury, and aflatoxin exposure (2). Despite remarkable advances in HCC treatment, the prognosis of this malignancy remains poor. The 5-year survival rate of HCC patients in the United States (3, 4) and Asia (5) is only 15–38%, largely attributed to late diagnosis, chemotherapy resistance, and a high incidence of tumor recurrence and metastasis.

Surgical resection, radiofrequency ablation, and transcatheter arterial chemoembolization (TACE) are effective treatments for localized HCC. Meanwhile, systemic therapy has provided new therapeutic options for patients with unresectable HCC (6). The advent of sorafenib, which prolonged survival in patients with unresectable HCC, ushered in a new era of systemic therapy with molecular-targeted agents (MTAs) for advanced HCC. Widely used multi-kinase inhibitors, including sorafenib, lenvatinib, regorafenib, and cabozantinib, as well as ramucirumab (a vascular endothelial growth factor [VEGF] inhibitor), have achieved satisfactory clinical outcomes (7–9). In addition to MTAs, immunotherapy based on immune checkpoint inhibitors (ICIs) has made substantial progress in recent years. Combination therapy with ICIs and VEGF inhibitors has demonstrated superior efficacy over sorafenib for advanced HCC (10). Currently, the combination of atezolizumab and bevacizumab has become a first-line standard regimen for patients with advanced HCC.

Liquid biopsy has emerged as a promising source of novel biomarkers in recent years and has been extensively applied in advanced HCC for monitoring tumor recurrence, evaluating treatment response, and detecting therapeutic resistance (11). Accordingly, an increasing number of studies have focused on identifying predictive biomarkers for response to systemic therapy, including immunotherapy and molecular targeted therapy, via liquid biopsy. The main components of liquid biopsy include circulating tumor DNA (ctDNA) (12), CTCs, various lymphocyte subpopulations (13), exosomes (14), and metabolites (15). Compared with traditional single-site tissue biopsy and serum protein biomarkers, liquid biopsy offers multiple advantages: high sensitivity and specificity, non-invasiveness, and the capability for dynamic real-time monitoring. Most importantly, it overcomes the limitations of tumor spatial and temporal heterogeneity (16). An increasing number of studies have investigated the role of CTCs as predictive biomarkers for immunotherapy response in various malignancies, including melanoma (17), non-small cell lung cancer (18), and breast cancer (19). In HCC, recent evidence suggests that CTC enumeration and molecular characterization may predict response to immune checkpoint inhibitors (20) and anti-angiogenic combination therapy. Studies have demonstrated that baseline CTC burden and dynamic CTC changes during treatment correlate with survival outcomes in lung cancer patients receiving systemic therapy (21). Furthermore, molecular profiling of CTCs has revealed that PD-L1 expression on CTCs and the presence of mesenchymal CTC subpopulations may serve as predictive biomarkers for immunotherapy efficacy (22). However, the clinical value of serial CTC monitoring in the context of combined immunotherapy and molecular targeted therapy for advanced HCC remains to be fully elucidated.

The primary aims of the present study were to explore the association between peripheral blood CTC levels and tumor treatment response, and to investigate the relationship between CTC counts and clinical prognosis in patients with advanced HCC receiving combined immunotherapy and molecular targeted therapy. Based on the rationale outlined above, we hypothesized that dynamic changes in peripheral blood CTC counts during the course of combined immunotherapy and molecular targeted therapy would correlate with radiological tumor response and survival outcomes in patients with advanced HCC, and that serial CTC monitoring could serve as a non-invasive liquid biopsy tool for early prediction of treatment efficacy and prognosis.

## Materials and methods

### Patients

From June 2019 to March 2022, a total of 125 consecutive patients with advanced HCC were enrolled at the Hepatic Surgery Center, Tongji Hospital, Tongji Medical College, Huazhong University of Science and Technology.

Inclusion criteria: (1) Age ≥ 18 years; (2) Clinical diagnosis of HCC confirmed by AFP detection and imaging examinations according to the American Association for the Study of Liver Diseases (AASLD) guidelines; (3) First-line treatment with combined immunotherapy and molecular targeted therapy; (4) Newly diagnosed HCC without prior anti-tumor treatment; (5) Liver function classified as Child-Pugh class A or B; (6) Eastern Cooperative Oncology Group (ECOG) performance status of 0 or 1.

Exclusion criteria: (1) History of prior anti-cancer treatment; (2) Incomplete follow-up data; (3) Poor adherence to prescribed medications.

This study was approved by the Ethics Committee of Tongji Medical College, Huazhong University of Science and Technology (Approval No. TJ-IRB20191101). All study procedures were performed in accordance with the Declaration of Helsinki. Written informed consent was obtained from all enrolled patients.

### Study design

All 125 patients received standard combined immunotherapy and molecular targeted therapy. Concurrent local treatments, including TACE and radiotherapy, were administered individually based on the 2019 Guidelines for the Diagnosis and Treatment of Primary Liver Cancer.

Peripheral blood samples (5 mL each) were collected at five time points: before treatment initiation, and 1 month, 3 months, 6 months, and 1 year after treatment. The ISET method was used to isolate and quantify CTCs. This technique relies on differences in cell size and deformability between tumor cells and normal blood cells to separate tumor cells via membrane filtration. Captured CTCs were further identified using specific cytochemical staining and standard histopathological and cytomorphological criteria under light microscopy (23, 24). An 8-µm-pore polycarbonate membrane equipped in the ISET system was used for blood filtration and CTC capture. All CTC enumeration on ISET filter membranes was performed by a single experienced cytopathologist who was blinded to patients’ clinical characteristics, treatment records, radiological response assessments, and follow-up outcomes. Samples were identified only by anonymized study IDs at the time of CTC identification and counting, ensuring unbiased morphological evaluation. A cell was defined as a CTC if it met at least four of the following six morphological criteria:

- Abnormal karyotypes (e.g., lobulated nuclei);
- Cell diameter > 15 µm;
- Irregular, indented, or wrinkled nuclear margins;
- Nuclear-to-cytoplasmic ratio > 0.8;
- Prominent giant nucleoli;
- Heterogeneous nuclear staining.

Cells with giant nucleoli or abnormal karyotypes plus at least two other criteria were also classified as CTCs.

### CTC detection

CTC detection was performed using the CTCBIOPSY^®^ detection system (Model: YZY-CTC-D100, Wuhan Youzhiyou Medical Technology Co., Ltd., China) (25). The filtration module and track-etched polycarbonate membranes (8 µm cylindrical pores) were provided by the manufacturer. The module contained 12 independent wells, allowing simultaneous filtration of 12 samples, with a filtration area of 0.6 cm² per well. Peripheral blood (5 mL) from HCC patients and healthy volunteers was collected in EDTA anticoagulant tubes. Blood samples were diluted 10-fold with filtration buffer containing 0.175% saponin, 0.2% paraformaldehyde, 0.0372% EDTA, and 0.1% bovine serum albumin. After a 10-minute incubation at room temperature, the diluted samples were loaded into the filtration wells. Each well received 10 mL of diluted blood (equivalent to 1 mL of original peripheral blood) and underwent gentle vacuum filtration.

After filtration, the membranes were rinsed once with phosphate-buffered saline (PBS), removed from the module, and air-dried. Areas corresponding to 1 mL of original blood were stained with hematoxylin and eosin (H&E) or May-Grünwald-Giemsa stain, rinsed with PBS, and dried. Microscopic examination was conducted on a dedicated stage without vacuum pressure.

### Immunotherapy and molecular targeted therapy

Molecular targeted agents: Sorafenib (26) (Nexavar^®^, Bayer, Germany) was administered orally at 400 mg twice daily. Lenvatinib (Lenvima^®^, Eisai, Tokyo, Japan) was given at a daily oral dose of 8 mg for patients weighing < 60 kg and 12 mg for patients weighing ≥ 60 kg (7).

Immune checkpoint inhibitors (ICIs): Camrelizumab (27) (SHR-1210, Jiangsu Hengrui Medicine Co., Ltd.), pembrolizumab (28) (KEYTRUDA^®^, Merck Sharp & Dohme Corp.), and tislelizumab (29) (BGB-A317, BeiGene) were used. The dosage and treatment course of ICIs followed current clinical guidelines and expert consensus.

All adverse reactions during treatment were manageable, and no patient discontinued treatment due to controllable adverse events. Specifically, all patients were initially treated at the recommended dose. In case of severe adverse events, the dose was reduced or the frequency was decreased as per the guidelines, but there was no interruption of the treatment. The specific treatment time, frequency, and occurrence of adverse events are shown in **Table 1**.

**Table 1:** Clinical characteristics of 125 patients with advanced HCC receiving combined immunotherapy and molecular targeted therapy.

Clinical characteristics	No. of patients
Age years, M (IQR)	56 (44-63)
Sex	
Male	101 (80.8%)
Female	24 (19.2%)
Child-Pugh score	
A	116 (92.8%)
B	9 (7.2%)
ECOG score	
0	84 (67.2%)
1	41 (32.8%)
Etiology	
HBV	101 (80.8%)
HCV	2 (1.6%)
Others	22 (17.6%)
Comorbidities	
Hypertension	
Yes	31 (24.8%)
No	94 (75.2%)
Diabetes mellitus	
Yes	15 (12.0%)
No	110 (88.0%)
Coronary heart disease	
Yes	6 (4.8%)
No	119 (95.2%)
Lifestyle factors	
Alcohol consumption	
Current	37 (29.6%)
Former	23 (18.4%)
Never	65 (52.0%)
Smoking history	
Current	42 (33.6%)
Former	20 (16.0%)
Never	63 (50.4%)
AFP (ng/mL)	
Positive	113 (90.4%)
Negative	12 (9.6%)
PIVKA-II	
Positive	118 (94.4%)
Negative	7 (5.6%)
Tumor diameter (cm)	7.1 (6.2-9.4)
Number of tumors	
Singe	19 (15.2%)
Multiple	106 (84.8%)
Portal vein tumor thrombosis	
No	58 (46.4%)
Yes	67 (53.6%)
BCLC stage	
B	36 (28.8%)
C	89 (71.2%)
CNLC stage	
IIb	36 (28.8%)
IIIa	67 (53.6%)
IIIb	22 (17.6%)
Extrahepatic metastasis	
Lung	10 (8%)
Bone	2 (1.6%)
Peritoneal cavity	1 (0.8%)
Multiple metastases	9 (7.2%)
No	103 (82.4%)
Combination treatments	
TACE alone	78 (62.4%)
TACE and RFA/MWA	12 (9.6%)
TACE and radiotherapy	35 (28.0)
Molecular targeted agents	
Lenvatinib	75 (60.0%)
Sorafenib	50 (40.0%)
Immune checkpoint inhibitors	
Camrelizumab	22 (17.6%)
Pembrolizumab	50 (40.0%)
Tislelizumab	53 (42.4%)
Treatment duration	
Targeted therapy (months), mean ± SD	13.5 ± 2.5
Immunotherapy cycles, mean ± SD	15 ± 1.5
Treatment-related adverse events	
Hand-foot syndrome	42 (33.6%)
Hypertension	38 (30.4%)
Liver function impairment	24 (19.2%)
Immune-related adverse events	28 (22.4%)
Discontinuation due to adverse events	0 (0%)

HCC, hepatocellular carcinoma; ECOG, Eastern Cooperative Oncology Group; HBV, hepatitis B virus; HCV, hepatitis C virus; AFP, alpha-fetoprotein; PIVKA-II, protein induced by vitamin K absence or antagonist-II; BCLC, Barcelona Clinic Liver Cancer; CNLC, China liver cancer staging; TACE, transcatheter arterial chemoembolization; RFA, radiofrequency ablation; MWA, microwave ablation; SD, Standard deviation.

### Study endpoints

The primary endpoint was tumor treatment response, which was categorized as complete response (CR), partial response (PR), stable disease (SD), and progressive disease (PD) according to mRECIST criteria (30). Objective response rate (ORR) was defined as the sum of CR and PR rates, and disease control rate (DCR) was defined as the sum of ORR and SD rates. Tumor response was independently assessed by two board-certified radiologists who were blinded to CTC results, laboratory data, and clinical outcomes. Discrepancies were resolved by consensus or by a third senior radiologist.

The secondary endpoints were progression-free survival (PFS) and overall survival (OS), which were used to analyze the correlation between CTC levels and patient prognosis. PFS was defined as the time from the initiation of systemic therapy to tumor progression or all-cause death. OS was defined as the time from treatment initiation to all-cause death. Survival data were censored at the last follow-up date.

Tumor progression was assessed by contrast-enhanced computed tomography (CT) or contrast-enhanced magnetic resonance imaging (MRI) every 3 months. The follow-up period ended in June 2023. Patient survival status was verified via official death registries and telephone follow-up.

### Statistical analysis

All statistical analyses were performed using IBM SPSS Statistics 22.0 (SPSS Inc., Chicago, USA). Statistical graphs were generated with GraphPad Prism 10. Continuous variables were presented as median and interquartile range (IQR), while categorical variables were expressed as counts and percentages. The Chi-square test or Mann-Whitney U test was used for between-group comparisons as appropriate. Repeated-measures analysis of variance (ANOVA) analysis was used to compare the CTC levels at different time points within the same group. Optimal CTC cut-off values at each time point were determined using receiver operating characteristic (ROC) curve analysis. Pairwise comparisons of AUCs between correlated ROC curves were performed using the DeLong test. The threshold maximizing the Youden index (sensitivity + specificity - 1) for predicting objective tumor response (CR + PR) was selected as the optimal cut-off. Kaplan-Meier survival curves were used to analyze the prognostic differences between groups, and the log-rank test was used to compare survival distributions. A two-sided P value < 0.05 was considered statistically significant.

## Results

### Baseline clinical characteristics of enrolled patients

The baseline clinical characteristics of the 125 patients with advanced HCC receiving combined immunotherapy and molecular targeted therapy are summarized in **Table 1**. The cohort was predominantly male (80.8%), and 80.8% of patients had chronic HBV infection. A total of 116 patients (92.8%) were classified as Child-Pugh class A, indicating well-preserved liver function. At baseline, 84 patients had an ECOG performance status of 0. Over half of the patients presented with portal vein tumor thrombosis (PVTT), and 71.2% were staged as Barcelona Clinic Liver Cancer (BCLC) stage C. Distant metastases were identified in 22 patients (17.6%) at treatment initiation, including lung metastases (8%), bone metastases (1.6%), peritoneal metastases (0.8%), and multiple organ metastases (7.2%) (**Table 1**).

All patients received combined local and systemic therapies. Among the 125 patients, 92 underwent TACE treatment, 7 received TACE combined with MWA treatment, 25 underwent TACE combined with radiotherapy, and 1 underwent TACE combined with MWA and radiotherapy (**Table 1**). The mean follow-up duration was 26 months (median: 24 months; range: 15–48 months). At the end of follow-up, 81 patients had died, corresponding to an overall mortality rate of 61.8%.

### CTC detection

A total of 461 peripheral blood samples were scheduled for collection. Blood samples could not be obtained from 75 patients at the 12-month follow-up, 42 at the 6-month time point, and 20 at the 3-month time point, mainly due to patient death and loss to follow-up. In addition, 27 samples were deemed non-compliant due to improper preservation.

The sample collection completeness rates at each time point were as follows: 100% (125/125) at baseline, 97.6% (122/125) at 1 month, 76.0% (95/125) at 3 months, 61.6% (77/125) at 6 months, and 32.8% (42/125) at 12 months (**Figure 1A**). The gradual decline in sample availability was attributed to patient mortality, loss to follow-up, and poor adherence to the study protocol.

**Figure 1:**
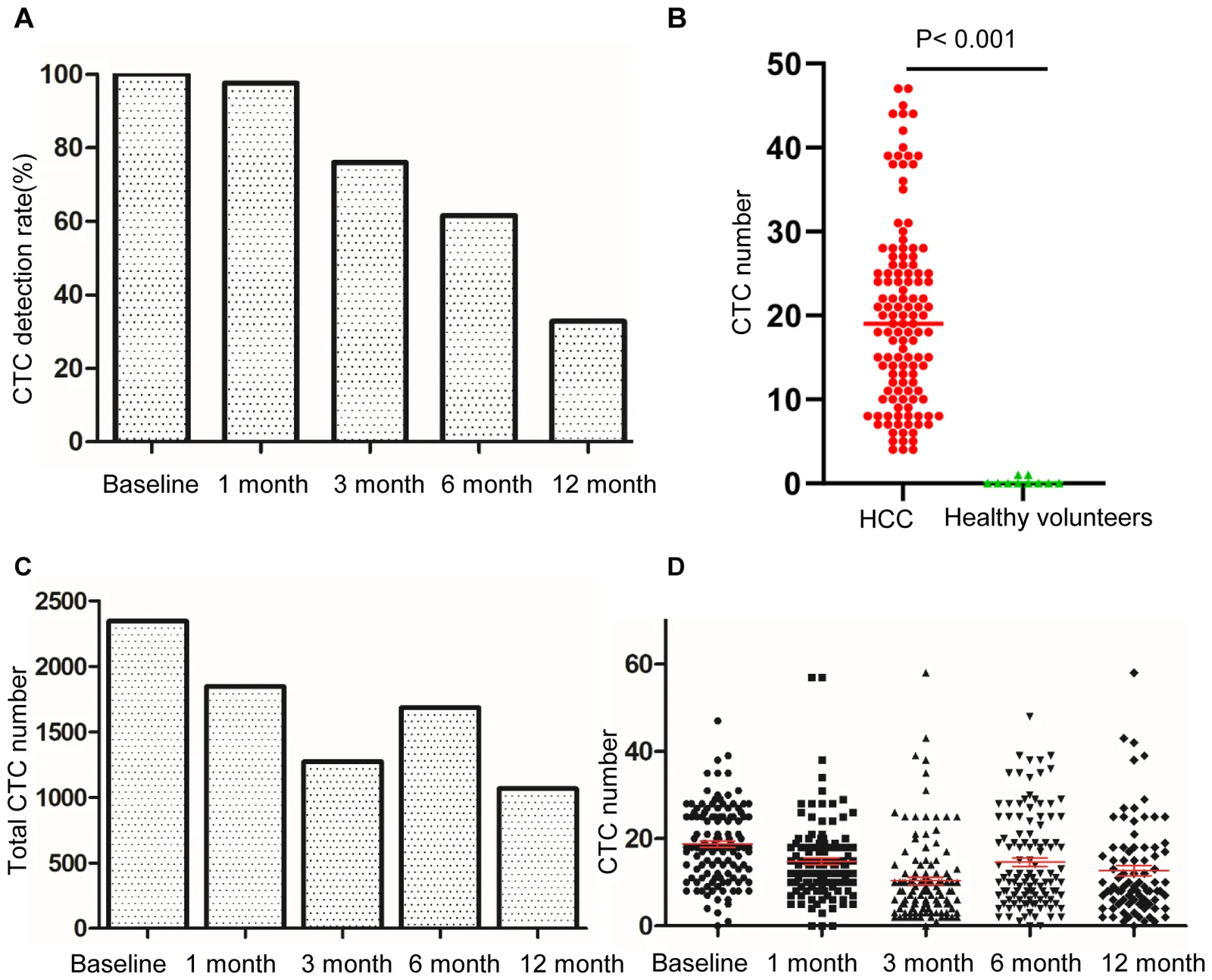
Dynamic detection of circulating tumor cells (CTCs) in peripheral blood of patients with advanced hepatocellular carcinoma (HCC). **(A)** Bar chart showing CTC detection rates at five time points: baseline, 1 month, 3 months, 6 months, and 12 months after treatment initiation. **(B)** Scatter plot comparing CTC counts between HCC patients (red dots) and healthy volunteers (green triangles), showing a statistically significant difference (P < 0.001). **(C)** Bar chart showing the total number of CTCs detected at each time point. **(D)** Scatter plot showing the distribution of CTC counts at each time point, with red horizontal lines indicating median values.

Ten healthy volunteers were enrolled to validate the performance of the ISET assay. CTCs were barely detectable in the peripheral blood of healthy individuals, and the CTC level differed significantly from that of HCC patients at baseline (P < 0.001; **Figure 1B**).

The total number of CTCs detected at each time point was 2,344 (baseline), 1,844 (1 month), 1,272 (3 months), 1,684 (6 months), and 1,065 (12 months) (**Figure 1C**). The median CTC counts (IQR) were 18.7 (12.0–25.0) at baseline, 14.8 (9.0–21.0) at 1 month, 10.3 (5.0–16.5) at 3 months, 14.6 (8.0–21.5) at 6 months, and 12.7 (6.0–19.5) at 12 months (**Figure 1D**). Overall, CTC levels initially decreased and then slightly rebounded, a trend consistent with effective anti-tumor treatment.

### Tumor treatment response

The waterfall plot in **Figure 2** illustrates the tumor response of all enrolled patients. Six patients (4.8%) achieved CR, 43 (33.6%) achieved PR, and 54 (43.2%) had SD. The overall DCR was 82.4% (103/125), while 22 patients (17.6%) developed PD. No patients discontinued treatment or died from adverse drug reactions. However, 14 patients (11.3%) were hospitalized for severe complications, including hand-foot syndrome, hypertension, and liver function impairment (**Table 1**).

**Figure 2:**
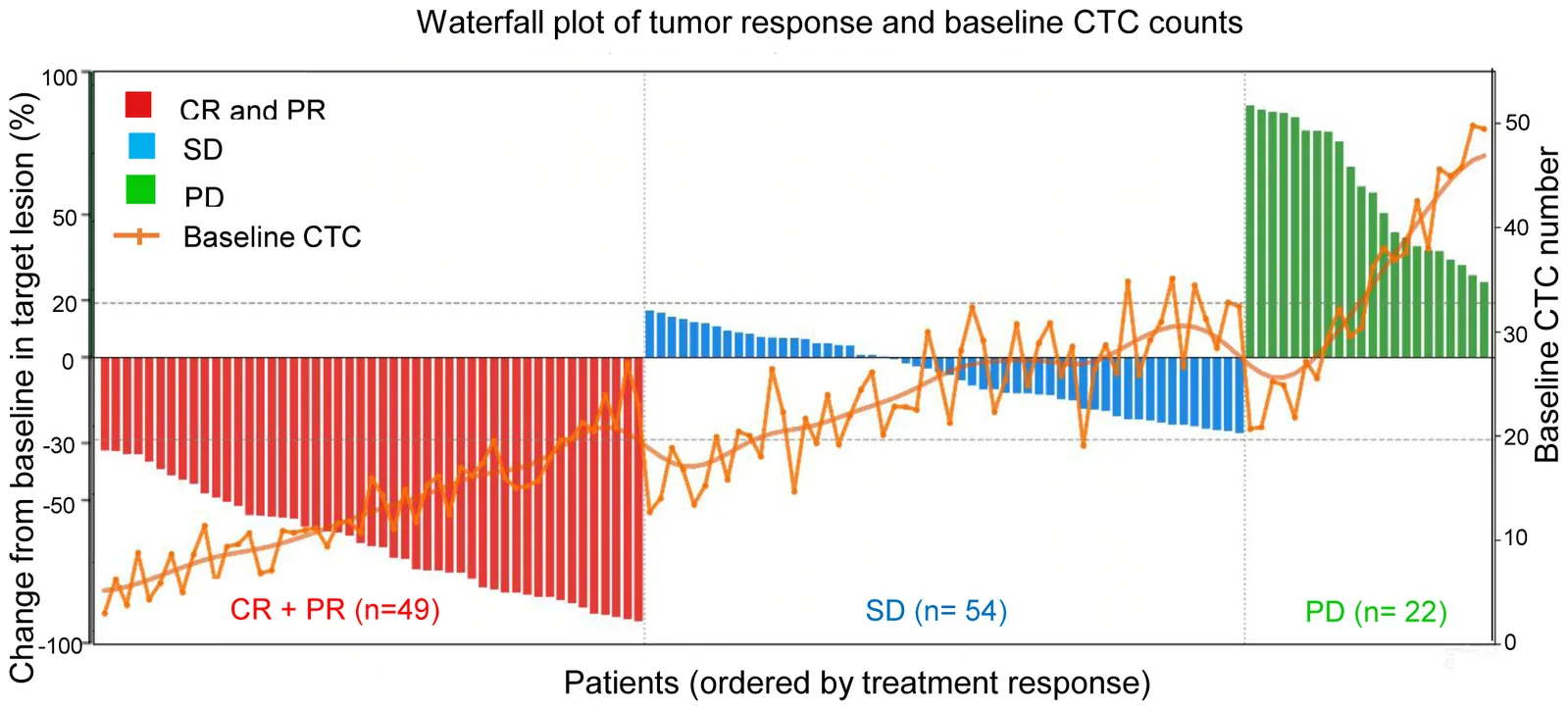
Waterfall plot of tumor response and baseline CTC counts. Percentage change from baseline in target lesion size (left y-axis) according to mRECIST criteria, sorted by treatment response: CR+PR (n = 49, red), SD (n = 54, blue), and PD (n = 22, green). The orange line (right y-axis) represents baseline CTC counts (/5 mL) for each patient, showing an increasing trend from CR/PR to PD. Dashed lines indicate mRECIST thresholds (+20% and −30%).

**Table 2** presents the clinical profiles and serial CTC counts of patients stratified by mRECIST treatment response. To explore longitudinal changes in CTC levels during treatment, we generated scatter plots for patients in the CR, PR, SD, and PD groups (**Figures 3A–D**).

**Table 2:** Clinical characteristics of patients grouped based on mRECIST criteria.

Clinical characteristics	CR (n=6)	PR (n=43)	SD (n=54)	PD (n=22)	P value
Age, years	59 ± 18	54 ± 8	56 ± 10	57 ± 12	H^†^ = 2.15; P = 0.542
Sex					
Male	6 (100%)	38 (88.4%)	42 (72.1%)	18 (81.8%)	χ² ^††^= 3.25; P = 0.354
Female	0 (0%)	5 (11.6%)	12 (27.9%)	7 (18.2%)	
Child-Pugh score					
A	6 (100%)	40 (93.0%)	51 (96.2%)	19 (86.4%)	χ² = 4.12; P = 0.249
B	0 (0%)	3 (7.0%)	3 (3.8%)	3 (13.6%)	
ECOG score					
0	5 (83.3%)	31 (72.1%)	34 (63.0%)	14 (63.6%)	χ² = 2.87; P = 0.412
1	1 (16.7%%)	12 (27.9%)	20 (37.0%)	8 (36.4%)	
Liver disease					
HBV	6 (100%)	33 (76.7%)	45 (83.3%)	17 (77.3%)	χ² = 5.63; P = 0.466
HCV	0 (0%)	1 (2.3%%)	1 (1.9%)	0 (0%)	
Others	0 (0%)	9 (21.0)	8 (14.8%)	5 (22.7%)	
AFP					
Positive	6 (100%)	38 (88.4%)	49 (90.7%)	20 (90.9%)	χ² = 1.25; P = 0.741
Negative	0 (0%)	5 (11.6%)	5 (9.3%)	2 (9.1%)	
BCLC stage					
B	1 (16.7%)	12 (27.9%)	16 (29.6%)	7 (31.8%)	χ² = 1.56; P = 0.669
C	5 (83.3%)	31 (72.1%)	38 (70.4%)	15 (68.2%)	
CNLC stage					
IIb	3 (50%)	11 (25.6%)	16 (29.6%)	6 (27.3%)	χ² = 8.34; P = 0.214
IIIa	1 (16.7%)	27 (62.8%)	29 (53.7%)	10 (45.4%)	
IIIb	2 (33.3%)	5 (11.6%)	9 (16.7%)	6 (27.3%)	
CTC counts before treatment, median (IQR)	16.0 (13.5–19.0)	17.0 (14.5–20.5)	24.0 (21.5–27.5)	27.0 (23.5–31.0)	H = 28.45; P = 0.011
CTC counts after 1 month, median (IQR)	15.5 (14.0–17.5)	15.5 (13.0–18.5)	22.5 (20.0–25.5)	26.0 (23.0–29.5)	H = 25.67; P = 0.024
CTC counts after 3 months, median (IQR)	9.0 (7.5–11.0)	11.0 (8.5–14.5)	21.5 (18.0–25.5)	28.0 (23.5–33.0)	H = 32.18; P = 0.003
CTC counts after 6 months, median (IQR)	7.0 (5.5–8.5)	11.5 (9.0–15.0)	20.0 (16.5–24.0)	28.5 (23.0–34.0)	H = 30.52; P = 0.027
CTC counts after 12 months, median (IQR)	8.5 (7.0–10.0)	12.0 (9.0–16.0)	17.0 (13.0–22.0)	–	H = 18.76; P = 0.034

HBV, hepatitis B virus; HCV, hepatitis C virus; ECOG, Eastern Cooperative Oncology Group; AFP, alpha-fetoprotein; BCLC, Barcelona Clinic Liver Cancer; CNLC, China liver cancer staging; CTC, circulating tumor cell; CR, complete response; PR, partial response; SD, stable disease; PD, progressive disease; IQR, Interquartile range.

^†^Kruskal-Wallis H test.

^††^Chi-square test.

**Figure 3:**
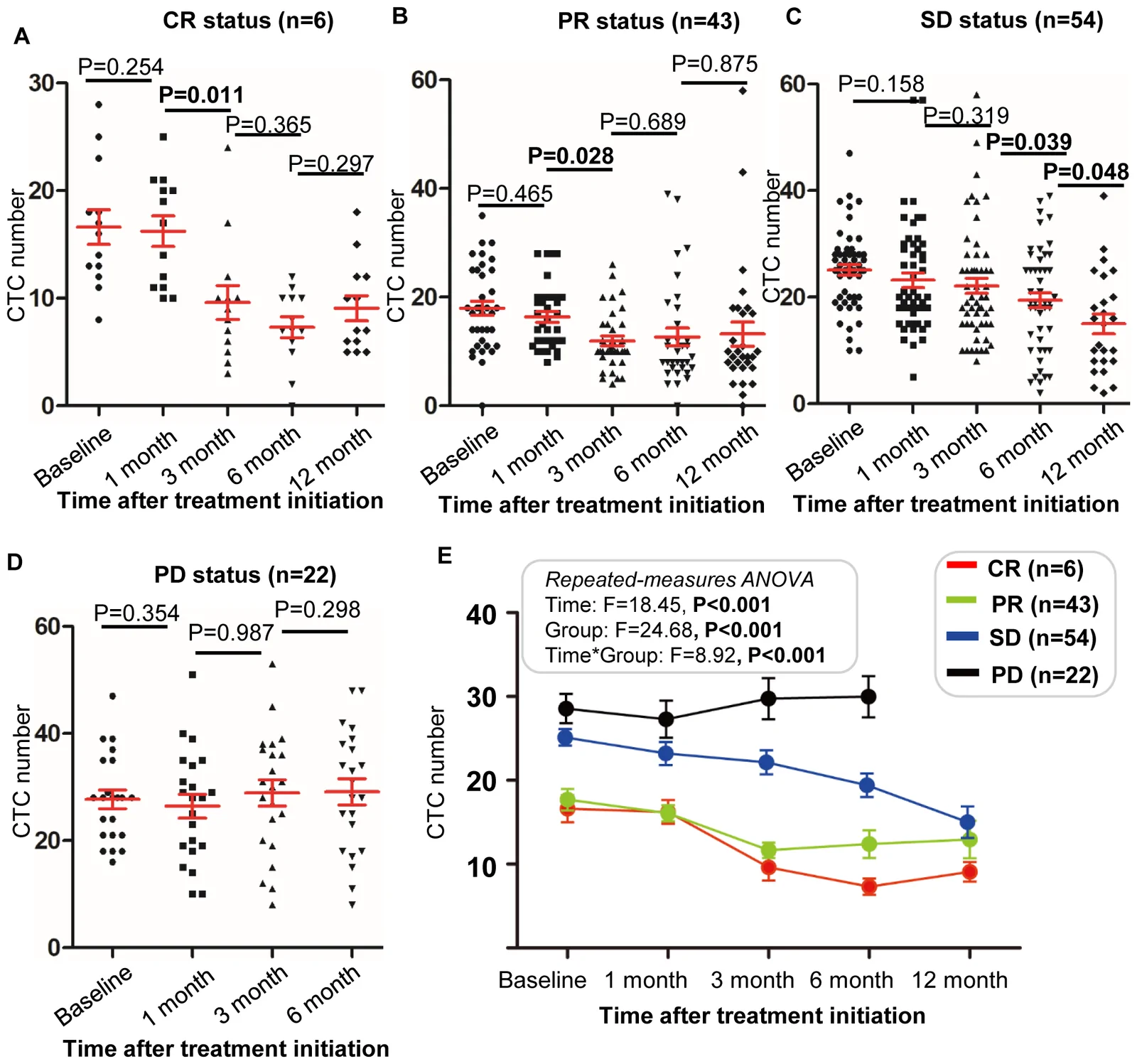
Serial CTC dynamics stratified by mRECIST treatment response. **(A–D)** Scatter plots of CTC counts at baseline and 1, 3, 6, and 12 months in CR (n = 6), PR (n = 43), SD (n = 54), and PD (n = 22) subgroups. Red bars denote median (IQR); *P* values from Wilcoxon signed-rank tests. **(E)** Median CTC trajectories across subgroups. Repeated-measures ANOVA: time F = 18.45, *P* < 0.001; group F = 24.68, *P* < 0.001; time × group F = 8.92, *P* < 0.001.

To investigate the differences in CTC levels at different treatment time points, we employed the Wilcoxon signed-rank test to compare the CTC variations between adjacent time points within each response group. For patients with CR, the median CTC counts (IQR) at baseline, 1 month, 3 months, 6 months, and 12 months post-treatment were 16.0 (13.5–19.0), 15.5 (14.0–17.5), 9.0 (7.5–11.0), 7.0 (5.5–8.5), and 8.5 (7.0–10.0), respectively. The corresponding P values for inter-timepoint comparisons were 0.254, 0.011, 0.365, and 0.297. For patients with PR, the sequential median CTC counts (IQR) were 17.0 (14.5–20.5), 15.5 (13.0–18.5), 11.0 (8.5–14.5), 11.5 (9.0–15.0), and 12.0 (9.0–16.0), with P values of 0.465, 0.028, 0.689, and 0.875. In the SD group, the median CTC counts (IQR) over time were 24.0 (21.5–27.5) at baseline, 22.5 (20.0–25.5) at 1 month, 21.5 (18.0–25.5) at 3 months, 20.0 (16.5–24.0) at 6 months, and 17.0 (13.0–22.0) at 12 months, and the associated P values were 0.158, 0.319, 0.039, and 0.048. For patients with PD, the median CTC counts (IQR) were 27.0 (23.5–31.0) at baseline, 26.0 (23.0–29.5) at 1 month, 28.0 (23.5–33.0) at 3 months, and 28.5 (23.0–34.0) at 6 months, with P values of 0.354, 0.987, and 0.298.

The line chart in **Figure 3E** summarizes the dynamic trends of CTC levels across the four groups. Next, to examine whether longitudinal CTC trajectories differed among patients with distinct treatment responses, repeated-measures analysis of variance (ANOVA) was performed with CTC count as the dependent variable, treatment response group (CR, PR, SD, PD) as the between-subjects factor, and time point (baseline, 1 month, 3 months, and 6 months) as the within-subjects factor. There was a significant main effect of time on CTC counts (F = 18.45, df = 2.68, 323.9, P < 0.001), indicating that peripheral blood CTC levels changed significantly over the course of systemic therapy. A significant between-group effect was also observed (F = 24.68, df = 3, 121, P < 0.001), demonstrating that overall CTC burden differed significantly among the four response groups. Most importantly, a significant time-by-group interaction effect was detected (F = 8.92, df = 8.03, 323.9, P < 0.001), indicating that the patterns of CTC change over time differed significantly according to treatment response. CTC counts in the CR and PR groups declined significantly from baseline to 3 months and remained low thereafter, whereas CTC levels in the PD group showed no significant reduction and remained persistently elevated throughout follow-up. The SD group exhibited a gradual decline in CTC counts starting at 3 months, though their overall CTC levels remained higher than those in the CR and PR groups.

### Predictive value of CTCs for treatment response

To evaluate the predictive value of CTC counts and AFP for objective ORR in advanced HCC, we constructed receiver ROC curves using CTC counts at baseline, 1 month, and 3 months post-treatment, as well as the combined indicators of CTCs and AFP. At baseline, CTC counts exhibited significantly better predictive performance than AFP (AUC = 0.841, 95% confidence interval [CI]: 0.772–0.910 vs. AUC = 0.692, 95% CI: 0.598–0.786; P = 0.015). With an optimal cut-off value of 15 cells/5 mL (determined by the Youden index), baseline CTC achieved a sensitivity of 69% and a specificity of 84%. The combined indicators of baseline CTC and AFP demonstrated the highest predictive accuracy (AUC = 0.965, 95% CI: 0.936–0.994), which was significantly superior to both AFP alone (P < 0.001) and CTC alone (P = 0.008), yielding a sensitivity of 95% and a specificity of 84% (**Figure 4A**). At 1 month post-treatment, neither CTC (AUC = 0.617, 95% CI: 0.515–0.719) nor AFP (AUC = 0.667, 95% CI: 0.568–0.766) showed satisfactory predictive performance, and no significant difference was observed between them (P = 0.423). The combined index yielded an AUC of 0.704 (95% CI: 0.608–0.800), which was not significantly higher than either biomarker alone (P = 0.512 vs. AFP; P = 0.178 vs. CTC). These findings suggest that CTC and AFP levels at 1 month post-treatment have limited value for early prediction of tumor response, likely due to the delayed onset of action of immunotherapy and molecular targeted therapy (**Figure 4B**). At 3 months post-treatment, both CTC (AUC = 0.906, 95% CI: 0.854–0.958) and AFP (AUC = 0.891, 95% CI: 0.835–0.947) demonstrated excellent predictive accuracy, with no significant difference between the two (P = 0.712). With an optimal cut-off of 10 cells/5 mL, 3-month CTC achieved a sensitivity of 80% and a specificity of 88%. The combined indicators at 3 months achieved the highest AUC of 0.971 (95% CI: 0.946–0.996), which was significantly superior to both AFP alone (P = 0.012) and CTC alone (P = 0.038), with a sensitivity of 95% and a specificity of 91% (**Figure 4C**).

**Figure 4:**
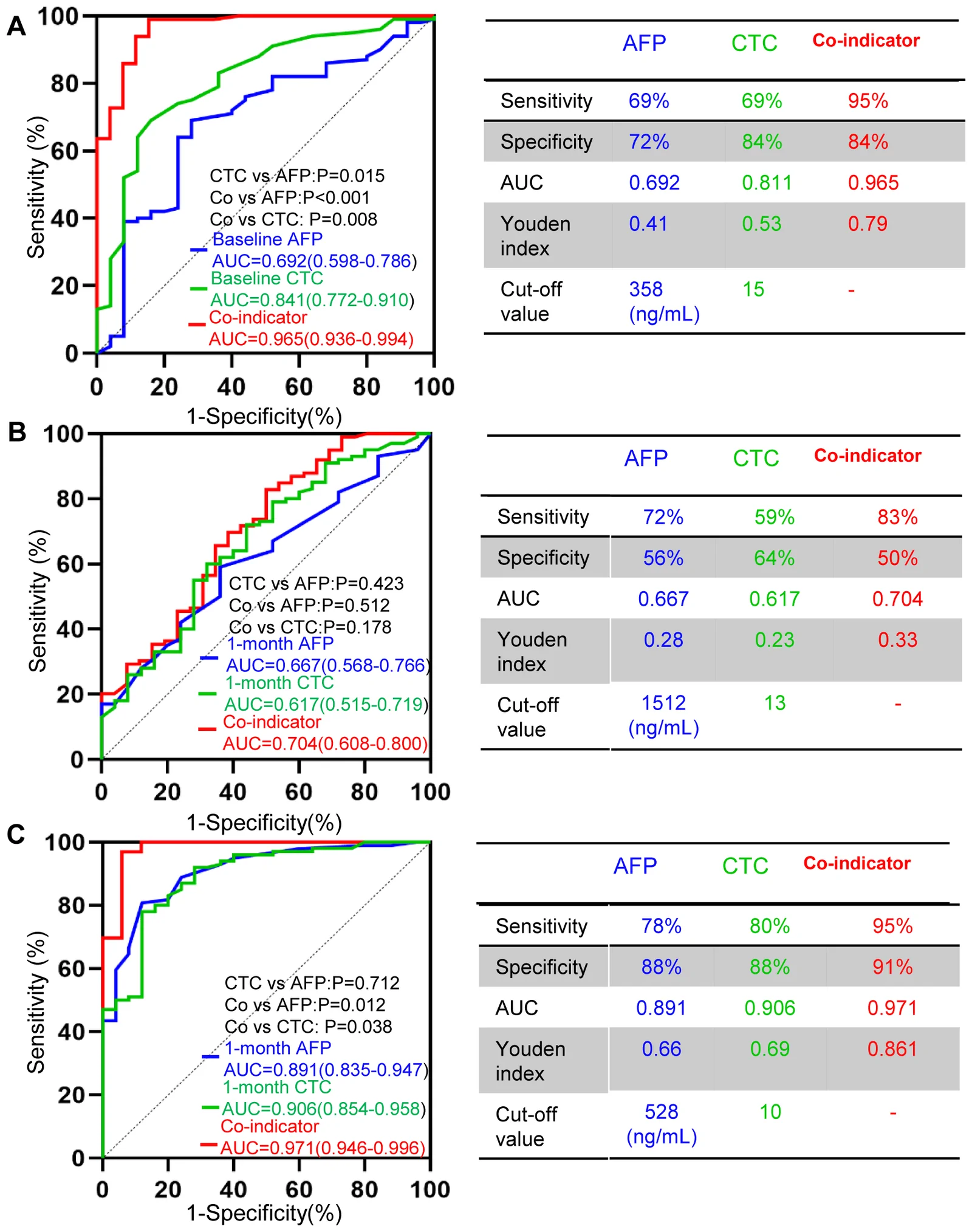
ROC analysis of AFP, CTC, and combined indicator for predicting treatment response. ROC curves at **(A)** baseline, **(B)** 1 month, and **(C)** 3 months. Red: AFP; green: CTC; blue: combined indicator. Adjacent tables report AUC, sensitivity, specificity, Youden index, and cut-off values. AUC comparisons by DeLong test. The combined indicator showed the highest predictive performance at baseline (AUC = 0.965) and 3 months (AUC = 0.971).

### CTCs and prognosis

All patients were stratified according to the optimal CTC cut-off values, and PFS and OS were compared between subgroups. Using 15 as the cut-off for baseline CTC counts, significant differences in both PFS and OS were observed between the two subgroups. Patients with baseline CTC counts < 15 had longer median PFS (not reached [95% CI: 18 months–not reached] vs. 7 months [95% CI: 5–10 months]) and median OS (not reached [95% CI: 22 months–not reached] vs. 12 months [95% CI: 9–16 months]) than those with baseline CTC counts ≥ 15 (log-rank P = 0.027 and P = 0.014, respectively; **Figures 5A, B**).

**Figure 5:**
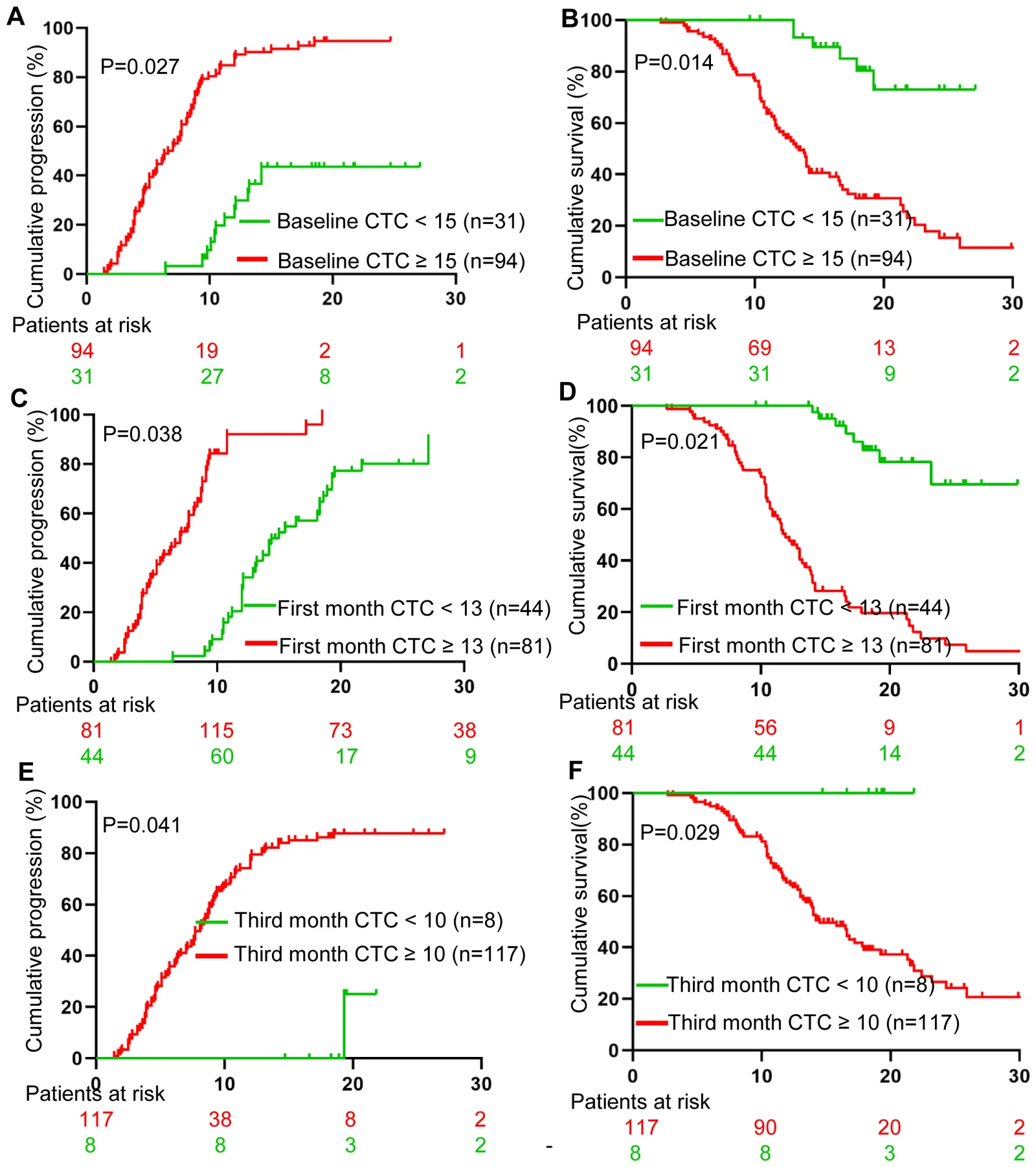
Kaplan–Meier survival curves stratified by CTC cut-off values. PFS **(A, C, E)** and OS **(B, D, F)** according to CTC cut-offs at baseline (15 cells), 1 month (13 cells), and 3 months (10 cells). Green: CTC below cut-off; red: CTC above cut-off. *P* values from log-rank tests are shown. Patient numbers at risk are displayed below each curve.

Patients with CTC counts < 13 at 1 month post-treatment also had superior median PFS (14 months [95% CI: 10–19 months] vs. 6 months [95% CI: 4–9 months], log-rank P = 0.038) and median OS (not reached [95% CI: 16 months–not reached] vs. 13 months [95% CI: 9–18 months], log-rank P = 0.021) compared with those with CTC counts ≥ 13 (**Figures 5C, D**).

Similarly, patients with CTC counts < 10 at 3 months post-treatment had longer median PFS (not reached [95% CI: 15 months–not reached] vs. 8 months [95% CI: 6–11 months], log-rank P = 0.041) and median OS (not reached [95% CI: 20 months–not reached] vs. 15 months [95% CI: 11–20 months], log-rank P = 0.021) than patients with CTC counts ≥ 10 (**Figures 5E, F**).

Univariate and multivariate Cox proportional-hazards regression analyses were performed to identify prognostic factors for PFS and OS. For PFS, multivariate analysis confirmed that BCLC stage C (HR = 1.78, 95% CI: 1.05–3.02, P = 0.032),PVTT (HR = 1.58, 95% CI: 1.21–3.04, P = 0.026), Baseline CTC ≥15 (HR = 2.16, 95% CI: 1.09–4.28, P = 0.023), CTC ≥ 10/5 mL at 3 months (HR = 1.85, 95% CI: 1.10–3.12, P = 0.020), and CTC ≥ 8/5 mL at 6 months (HR = 2.05, 95% CI: 1.88–2.56, P = 0.011) were independent prognostic factors for PFS (**Table 3**). For OS, multivariate analysis demonstrated that BCLC stage C (HR = 1.85, 95% CI: 1.08–3.17, P = 0.025), PVTT (HR = 2.36, 95% CI: 1.69–4.87, P = 0.015), baseline AFP ≥ 400 ng/mL (HR = 1.70, 95% CI: 1.05–2.75, P = 0.030), Baseline CTC ≥15 (HR = 1.25, 95% CI: 1.08–1.92, P = 0.033), and CTC ≥ 10/5 mL at 3 months (HR = 1.92, 95% CI: 1.13–3.27, P = 0.016) were independent prognostic factors for OS (**Table 4**).

**Table 3:** Univariate and multivariate Cox regression analyses for progression-free survival (PFS).

Variables	Univariate HR (95% CI)	P value	Multivariate HR (95% CI)	P value
Sex (Male vs. Female)	1.12 (0.68–1.85)	0.652	1.05 (0.58–1.90)	0.873
Age (≥60 vs. <60 yr)	1.05 (0.71–1.56)	0.803	0.98 (0.62–1.55)	0.932
HBsAg (Positive vs. Negative)	1.18 (0.72–1.93)	0.514	1.08 (0.60–1.94)	0.791
Liver cirrhosis (Yes vs. No)	1.21 (0.78–1.88)	0.402	1.10 (0.65–1.86)	0.723
Child-Pugh grade (B vs. A)	1.35 (0.76–2.40)	0.301	1.15 (0.58–2.28)	0.689
ECOG performance status(0 vs 1)	0.87 (0.68-1.52)	0.215	0.95 (0.89-1.36)	0.292
Concomitant local therapy (Yes vs. No)	0.82 (0.53–0.97)	**0.011**	0.75 (0.45–1.25)	0.271
Tumor stage (BCLC C vs. BCLC B)	2.15 (1.38–3.35)	**<0.001**	1.78 (1.05–3.02)	**0.032**
PVTT(Yes vs. No)	2.54 (2.01-4.35)	**<0.001**	1.58 (1.21-3.04)	**0.026**
Baseline AFP (≥400 vs. <400 ng/mL)	1.78 (1.18–2.69)	**0.006**	1.12 (0.98–2.60)	0.445
Baseline CTC (≥15 vs. <15 /5 mL)	1.85 (1.22–2.81)	**0.004**	2.16 (1.09–4.28)	**0.023**
CTC at 1 month (≥13 vs. <13 /5 mL)	1.42 (0.93–2.17)	**0.105**	1.15 (0.68–1.95)	0.602
CTC at 3 months (≥10 vs. <10 /5 mL)	2.05 (1.32–3.18)	**0.001**	1.85 (1.10–3.12)	**0.020**
CTC at 6 months (≥8 vs. <8 /5 mL)	2.28 (1.42–3.66)	**<0.001**	2.05 (1.88–2.56)	**0.011**
CTC at 12 months (≥5 vs. <5 /5 mL)	1.38 (0.82–2.32)	0.224	1.12 (0.60–2.09)	0.721

HR, hazard ratio; CI, confidence interval; AFP, alpha-fetoprotein; ECOG, Eastern Cooperative Oncology Group; PVTT, portal vein tumor thrombus; CTC, circulating tumor cell. Cox proportional-hazards regression was used. The multivariate model included all 13 variables listed above. Bold P values indicate statistical significance (P < 0.05).

**Table 4:** Univariate and multivariate Cox regression analyses for overall survival (OS).

Variables	Univariate HR (95% CI)	P value	Multivariate HR (95% CI)	P value
Sex (Male vs. Female)	1.08 (0.65–1.80)	0.763	1.02 (0.56–1.86)	0.941
Age (≥60 vs. <60 yr)	1.10 (0.73–1.66)	0.642	1.03 (0.64–1.65)	0.901
HBsAg (Positive vs. Negative)	1.15 (0.69–1.92)	0.591	1.05 (0.58–1.90)	0.872
Liver cirrhosis (Yes vs. No)	1.25 (0.79–1.98)	0.341	1.12 (0.65–1.93)	0.683
Child-Pugh grade (B vs. A)	1.42 (0.79–2.55)	0.241	1.20 (0.60–2.40)	0.612
ECOG performance status(0 vs 1)	0.92 (071-1.32)	0.369	0.92 (0.79-1.59)	0.362
Concomitant local therapy (Yes vs. No)	0.78 (0.50–0.82)	0.021	0.72 (0.43–1.21)	0.213
Tumor stage (BCLC C vs. BCLC B)	2.28 (1.44–3.61)	**<0.001**	1.85 (1.08–3.17)	0.025
PVTT(Yes vs. No)	3.25 (2.96-5.32)	**<0.001**	2.36 (1.69-4.87)	0.015
Baseline AFP (≥400 vs. <400 ng/mL)	1.88 (1.23–2.87)	0.003	1.70 (1.05–2.75)	0.030
Baseline CTC (≥15 vs. <15 /5 mL)	1.95 (1.27–3.00)	0.002	1.25 (1.08–1.92)	0.033
CTC at 1 month (≥13 vs. <13 /5 mL)	1.48 (0.96–2.28)	0.075	1.18 (0.69–2.02)	0.543
CTC at 3 months (≥10 vs. <10 /5 mL)	2.18 (1.39–3.42)	**<0.001**	1.92 (1.13–3.27)	0.016
CTC at 6 months (≥8 vs. <8 /5 mL)	2.42 (1.49–3.93)	**<0.001**	1.15 (0.97–2.79)	0.108
CTC at 12 months (≥5 vs. <5 /5 mL)	1.45 (0.85–2.47)	0.171	1.18 (0.62–2.24)	0.611

HR, hazard ratio; CI, confidence interval; AFP, alpha-fetoprotein; ECOG, Eastern Cooperative Oncology Group; PVTT, portal vein tumor thrombus; CTC, circulating tumor cell. Cox proportional-hazards regression was used. The multivariate model included all 13 variables listed above. Bold P values indicate statistical significance (P < 0.05).

### Representative cases

#### Case 1

**Figure 6** presents contrast-enhanced computed tomography (CT) images of a 61-year-old male patient with multifocal HCC located in the left inner hepatic lobe. The patient received TACE combined with radiofrequency ablation (RFA), followed by sequential immunotherapy and molecular targeted therapy. At baseline, arterial, portal venous, and delayed phase CT scans were acquired, and a total of 5 CTCs were detected in his peripheral blood (**Figure 6A**).

**Figure 6:**
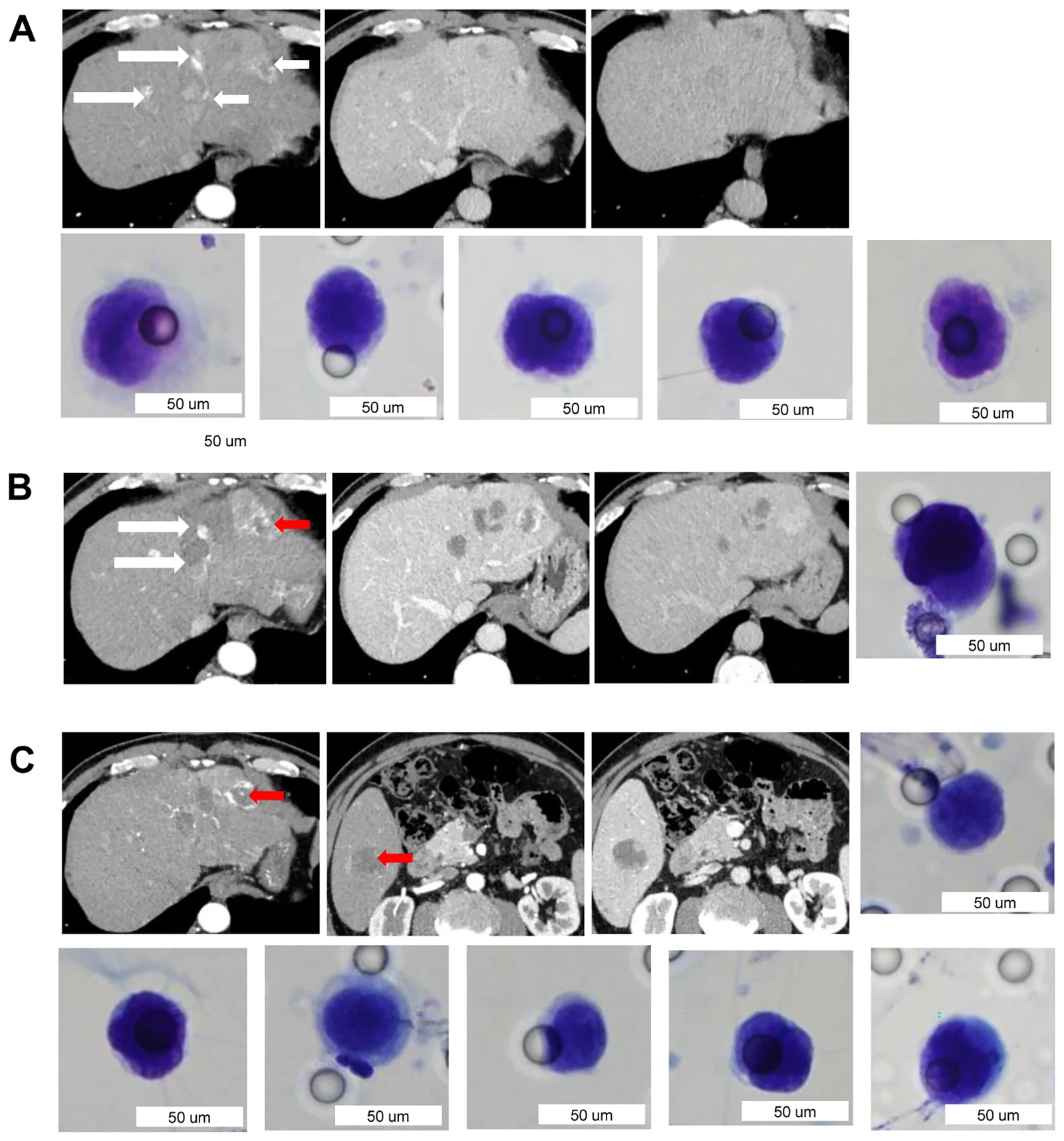
Representative case 1: A 61-year-old male patient with multifocal hepatocellular carcinoma. **(A)** Baseline contrast-enhanced CT images (arterial, portal venous, and delayed phases) showing multifocal HCC lesions in the left inner hepatic lobe (white arrows). Corresponding CTC morphology (H&E staining) showing 5 CTCs detected at baseline. **(B)** Three-month follow-up CT images showing partial response to treatment. CTC morphology showing only 1 CTC detected, indicating a marked reduction from baseline. **(C)** Six-month follow-up CT images showing tumor progression: new enhancing lesions in the left outer lobe (red arrows). CTC morphology showing increased CTC counts corresponding to disease progression. Scale bar: 50 μm for all CTC micrographs.

At 3 months post-treatment, follow-up contrast-enhanced CT was performed, and only one CTC was detected — a marked reduction from the baseline level (**Figure 6B**). At the 6-month follow-up, imaging confirmed tumor progression: lesions in the left outer lobe showed obvious enhancement in the arterial phase, accompanied by a rise in peripheral blood CTC counts (**Figure 6C**).

#### Case 2

**Figure 7** shows baseline contrast-enhanced CT images of a 67-year-old male patient with diffuse multifocal HCC, which occupied most of the liver parenchyma. No extrahepatic metastasis was detected at enrollment. The patient was treated with TACE plus sequential immunotherapy and molecular targeted therapy. Baseline arterial, portal venous, and delayed phase CT scans revealed tumor lesions, and 5 CTCs were detected in the peripheral blood (**Figure 7A**).

**Figure 7:**
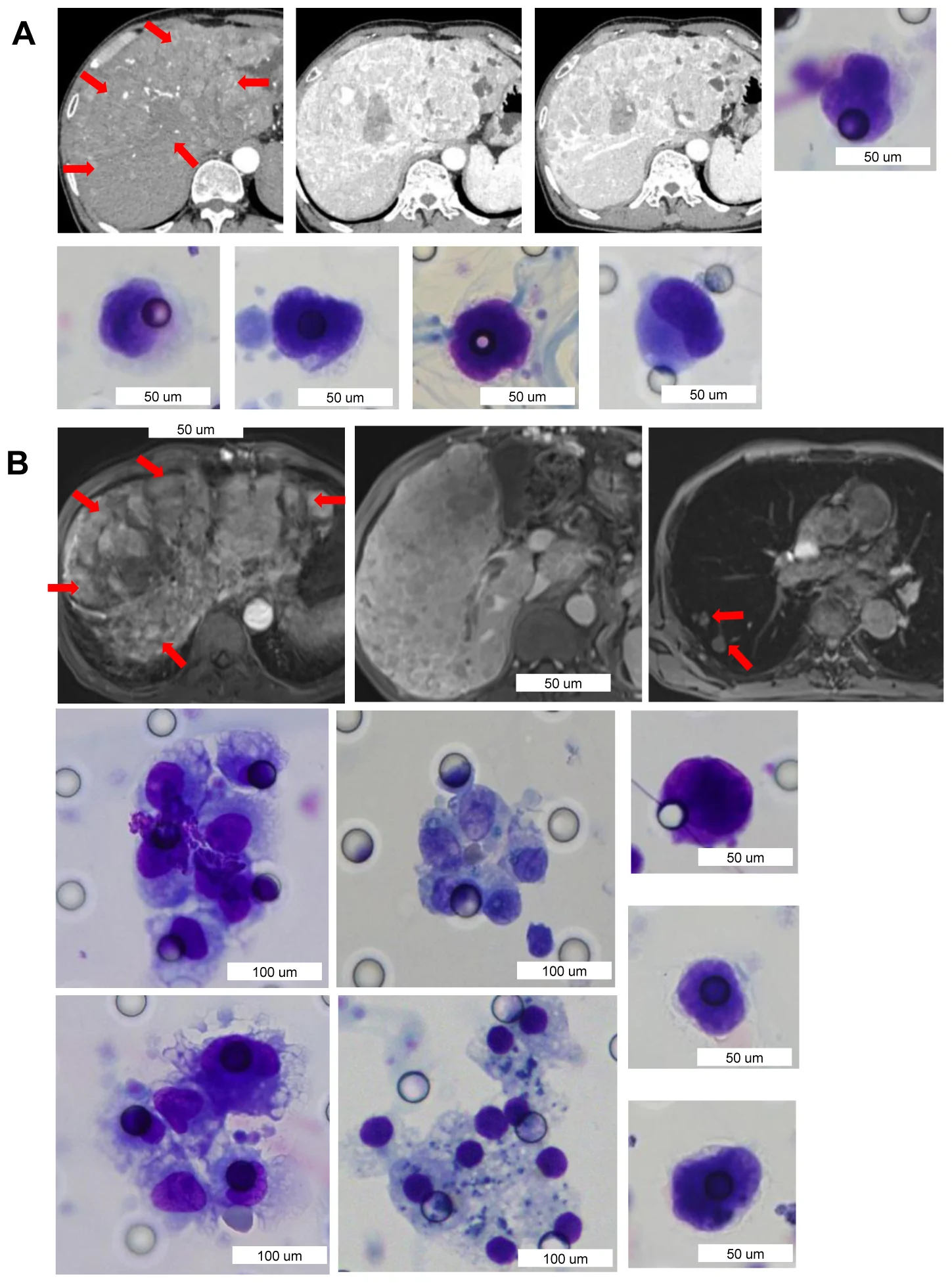
Representative case 2: A 67-year-old male patient with diffuse multifocal hepatocellular carcinoma. **(A)** Baseline contrast-enhanced CT images (arterial, portal venous, and delayed phases) showing diffuse multifocal HCC occupying most of the liver parenchyma (red arrows). Corresponding CTC morphology (H&E staining) showing 5 individual CTCs detected at baseline. **(B)** Three-month follow-up CT images showing progressive disease: enlarged hepatic lesions without necrosis and new lung metastases (red arrows). CTC morphology showing substantially increased CTC counts and the presence of circulating tumor microemboli (CTM, defined as clusters of ≥3 tumor cells). Scale bars: 50 μm for single CTCs, 100 μm for CTM clusters.

At 3 months post-treatment, imaging showed progressive disease: the hepatic lesions enlarged without necrosis, and new lung metastases developed. Circulating tumor microemboli (CTM) were defined as cell clusters consisting of three or more tumor cells. Peripheral blood CTC counts increased substantially, and CTM were also identified (**Figure 7B**).

## Discussion

In this real-world retrospective study of 125 patients with advanced HCC receiving combined immunotherapy and molecular targeted therapy, we demonstrated that dynamic changes in peripheral blood CTC counts, detected by the size-based ISET platform, were closely correlated with radiological tumor response assessed by mRECIST criteria. Patients achieving CR or PR exhibited a progressive and sustained decline in CTC counts, whereas those with PD maintained persistently elevated CTC levels throughout treatment. Patients with SD showed an intermediate pattern, with CTC counts beginning to decline at 3 months but remaining higher than those in the CR/PR groups. Furthermore, baseline CTC counts ≥15 cells/5 mL, 1-month CTC ≥13 cells/5 mL, and 3-month CTC ≥10 cells/5 mL were associated with significantly shorter PFS and OS. Multivariable Cox regression analyses confirmed that tumor stage, CTC counts at 3 months and 6 months, and baseline AFP were independent prognostic factors for PFS and/or OS. ROC analysis demonstrated that baseline and 3-month CTC counts, particularly in combination with AFP, exhibited excellent predictive accuracy for objective tumor response, whereas 1-month CTC levels had limited predictive value.

Previous studies have mainly focused on the value of CTCs in the diagnosis, prognosis assessment, and monitoring of recurrence of HCC (31). This study takes advantage of the dynamic monitoring capabilities of liquid biopsy and innovatively introduces CTCs for the monitoring of the systemic treatment effect of advanced HCC. On one hand, our study is among the first to systematically monitor CTC dynamics at five serial time points (baseline, 1 month, 3 months, 6 months, and 12 months) during systemic therapy, enabling characterization of CTC trajectories and their correlation with treatment response over time. On the other hand, our study specifically addresses the growing population of patients with unresectable advanced HCC receiving first-line combined immunotherapy and molecular targeted therapy, a clinical scenario in which CTC monitoring has not been extensively investigated. Furthermore, we demonstrated that the combination of CTC and AFP outperforms either biomarker alone for predicting objective tumor response, particularly at baseline and 3 months post-treatment, supporting a multi-analyte liquid biopsy approach.

Our findings are consistent with previous studies investigating the prognostic value of CTCs in HCC. Ye et al. (32) reported that elevated postoperative CTC counts (>2 and >5 cells) and perioperative CTC dynamics were strongly associated with PFS in HBV-related HCC patients undergoing hepatectomy, serving as more reliable prognostic markers than single time-point measurements. Similarly, Zhou et al. (33) demonstrated that preoperative CTC counts could predict microvascular invasion and that dynamic CTC monitoring indicated prognosis in HCC patients. In line with these reports, our study extends the evidence to the setting of advanced HCC undergoing systemic therapy, confirming that serial CTC monitoring provides superior prognostic information compared with a single baseline measurement.

Systemic therapy constitutes a cornerstone of treatment for advanced HCC. In the present study, persistently elevated CTC levels were observed in patients experiencing tumor progression, and the majority of these patients ultimately succumbed to disease. Tumor progression represents the leading cause of death among patients with advanced HCC. Despite targeted-immunotherapy combination regimens, tumor progression remains an inevitable clinical event, which poses substantial challenges for clinical management. Tumor epithelial-mesenchymal transition (EMT), immune evasion and metastatic dissemination seem to be the key factors contributing to tumor progression.

The dynamic changes in peripheral blood CTC counts observed in our study likely reflect complex biological processes underlying tumor response to systemic therapy. A key mechanism involves EMT, a process by which epithelial tumor cells acquire mesenchymal phenotypes with enhanced migratory capacity, invasiveness, and resistance to apoptosis (34). Under the selective pressure of anti-angiogenic targeted agents and immune checkpoint inhibitors, tumor cells may undergo EMT, resulting in downregulation of epithelial markers such as EpCAM and cytokeratins, and upregulation of mesenchymal markers including vimentin and N-cadherin (35). Importantly, the ISET platform employed in our study isolates CTCs based on cell size and deformability rather than EpCAM expression, thereby enabling capture of both epithelial and mesenchymal-like CTC subpopulations. This EpCAM-independent approach may partially explain the robust dynamic CTC changes observed across treatment response groups. In patients achieving CR or PR, the progressive decline in CTC counts may reflect the elimination of therapy-sensitive epithelial CTCs; conversely, persistently elevated or rising CTC levels in the PD group may indicate the emergence of EMT-mediated resistant clones that survive systemic therapy and contribute to disease progression.

Another critical mechanism underlying the association between CTC dynamics and treatment response involves immune evasion. CTCs employ multiple strategies to survive in the hostile circulatory environment, including downregulation of major histocompatibility complex (MHC) class I molecules, expression of immune checkpoint ligands such as programmed death-ligand 1 (PD-L1), and recruitment of immunosuppressive platelets, neutrophils, and regulatory T cells (36). These mechanisms enable CTCs to evade natural killer (NK) cell and T-cell-mediated immune surveillance (37). In the context of immune checkpoint inhibitor therapy, restoration of anti-tumor immunity may enhance the clearance of CTCs, which could account for the declining CTC trajectories observed in responders. Conversely, in patients with progressive disease, CTCs may exploit immune evasion mechanisms to persist in the circulation despite treatment, leading to sustained high CTC levels.

Furthermore, the strong correlation between baseline CTC burden and treatment response suggests that higher pre-treatment CTC levels reflect greater tumor aggressiveness and metastatic potential, which may explain the inferior outcomes observed in patients with elevated baseline CTC counts. The presence of CTC clusters, also termed circulating tumor microemboli (CTM), represents a particularly ominous biological phenomenon. Compared with single CTCs, CTC clusters possess 20- to 50-fold greater metastatic potential, attributed to enhanced resistance to anoikis, cooperative cell-cell signaling, and mechanical protection from shear stress and immune attack in the circulation (38, 39). In our study, CTM were identified in patients with progressive disease (as illustrated in Case 2), but not in those achieving CR or PR, suggesting that the emergence of CTC clusters may signify a more aggressive tumor phenotype and higher risk of metastatic dissemination.

From a clinical perspective, our findings support the integration of serial CTC monitoring into the management of advanced HCC patients receiving systemic therapy. The observation that CTC declines become significant at 3 months post-treatment—aligning with the typical timing of radiological response assessment—suggests that CTC dynamics could serve as an early surrogate marker for treatment efficacy, potentially enabling timely therapeutic modifications before radiological progression becomes evident. The combination of CTC and AFP demonstrated superior predictive performance over either biomarker alone, highlighting the value of multi-analyte liquid biopsy approaches. Given that tissue biopsy in advanced HCC is often challenging and carries bleeding risk, non-invasive CTC monitoring offers a safe and repeatable alternative for real-time assessment of tumor biology and treatment response.

Several limitations of this study should be acknowledged. First, this was a single-center, real-world retrospective observational study rather than a randomized controlled trial, and therefore the potential confounding effects of heterogeneous systemic or local treatment modalities on CTC dynamics and clinical outcomes cannot be completely eliminated. Although all patients received first-line combined immunotherapy and molecular targeted therapy as the systemic backbone, concomitant local-regional therapies—including transcatheter arterial chemoembolization (TACE, 73.6%), radiotherapy (20.0%), and microwave ablation (5.6%)—were administered at the discretion of treating physicians. While we attempted to mitigate this confounding through multivariable Cox regression adjustment and subgroup analyses, residual confounding by unmeasured factors or specific drug regimens cannot be fully excluded. Future prospective studies with standardized treatment protocols are warranted.

Second, CTC identification was based exclusively on cytomorphological criteria after H&E or May-Grünwald-Giemsa staining, without immunocytochemical confirmation using epithelial markers (EpCAM, cytokeratins) and leukocyte markers (CD45). This approach carries inherent limitations, including potential false-positive identification of large activated leukocytes or endothelial cells, and false-negative results for small or de-differentiated tumor cells. Immunocytochemical staining was not feasible because the ISET filter samples were processed initially for routine cytopathological evaluation and were no longer available for additional staining due to sample depletion. Future prospective studies should incorporate immunophenotypic validation alongside morphological criteria.

Third, substantial sample attrition occurred during follow-up, with CTC sample availability declining from 100% at baseline to 32.8% at 12 months, primarily due to patient death, loss to follow-up, and sample non-compliance. This attrition may introduce survivorship bias, particularly for the 6- and 12-month data, which reflect highly selected survivor cohorts. Nevertheless, the late time-point data should be interpreted with caution.

Fourth, the majority of enrolled patients had HBV-related HCC (80.8%), and the efficacy of immunotherapy may differ in patients with non-viral HCC. Therefore, the generalizability of our findings to non-HBV, non-HCV HCC populations may be limited. Finally, the sample size of 125 patients, while adequate for exploratory analysis, may be insufficient for robust subgroup analyses, particularly for the CR group (n=6).

In conclusion, our study demonstrates that dynamic changes in peripheral blood CTC counts, detected by the size-based ISET platform, are closely correlated with tumor treatment response and survival outcomes in patients with advanced HCC receiving combined immunotherapy and molecular targeted therapy. Baseline and 3-month CTC counts, particularly in combination with AFP, serve as reliable predictive and prognostic biomarkers. The biological rationale—encompassing EMT-mediated phenotypic plasticity, immune evasion, ongoing metastatic dissemination, and CTC cluster formation—supports the clinical utility of serial CTC monitoring as a non-invasive liquid biopsy tool for real-time assessment of therapeutic efficacy. Further large-scale, multicenter prospective studies with standardized treatment protocols and immunophenotypic CTC validation are warranted to confirm our findings.

## Data Availability

The datasets presented in this study can be found in online repositories. The names of the repository/repositories and accession number(s) can be found below: The dataset(s) supporting the conclusions of this article is available in the (figure) repository, (10.6084/m9.figshare.20499261).

## Glossary

HCC: hepatocellular carcinoma
CTC: circulating tumor cell
ISET: isolation by size of epithelial tumor cells
mRECIST: modified Response Evaluation Criteria in Solid Tumors
CR: complete response
PR: partial response
SD: stable disease
PD: progressive disease
ORR: objective response rate
DCR: disease control rate
PFS: progression-free survival
OS: overall survival
ROC: receiver operating characteristic
AFP: alpha-fetoprotein
PIVKA-II: protein induced by vitamin K absence or antagonist-II
TACE: transcatheter arterial chemoembolization
ICI: immune checkpoint inhibitor
MTA: molecular targeted agent
VEGF: vascular endothelial growth factor
TME: tumor microenvironment
ctDNA: circulating tumor DNA
ECOG: Eastern Cooperative Oncology Group
BCLC: Barcelona Clinic Liver Cancer
PVTT: portal vein tumor thrombosis
CT: computed tomography
MRI: magnetic resonance imaging
H&E: hematoxylin and eosin
PBS: phosphate-buffered saline
CTM: circulating tumor microemboli
EMT: epithelial-mesenchymal transition
HBV: hepatitis B virus
HCV: hepatitis C virus
NASH: non-alcoholic steatohepatitis
RFA: radiofrequency ablation
MWA: microwave ablation
AASLD: American Association for the Study of Liver Diseases
EDTA: ethylenediaminetetraacetic acid
AUC: area under the curve
CI: confidence interval
HR: hazard ratio
IQR: interquartile range
ANOVA: analysis of variance
MHC: major histocompatibility complex
PD-L1: programmed death-ligand 1
NK: natural killer
CNLC: China Liver Cancer staging
Child-Pugh: Child-Pugh liver function classification
SD (stat): standard deviation
